# A Case of Human Trafficking in Appalachia and What Emergency Physicians Can Learn from It

**DOI:** 10.5811/westjem.58400

**Published:** 2023-05-05

**Authors:** Kelli L. Jarrell, Christa Pulvino, Alexis Kimmel, Bailee Stark, Harmanjit Khokhar, Laura Janneck, Sally A. Santen

**Affiliations:** * University of Cincinnati, Department of Emergency Medicine, Cincinnati, Ohio; † HCA St. Lucie Medical Center, Department of Emergency Medicine, Port St. Lucie, Florida; ‡ University of Oklahoma School of Community Medicine, Department of Emergency Medicine, Tulsa, Oklahoma

## Abstract

Human trafficking is an ongoing, global human rights crisis and one of the largest illicit industries worldwide. Although there are thousands of victims identified each year within the United States, the true extent of this problem remains unknown due to the paucity of data. Many victims seek care in the emergency department (ED) while being trafficked but are often not identified by clinicians due to lack of knowledge or misconceptions about trafficking. We present a case of an ED patient being trafficked in Appalachia as an educational stimulus and discuss several unique aspects of trafficking in rural communities, including lack of awareness, prevalence of familial trafficking, high rates of poverty and substance use, cultural differences, and a complex highway network system. The lack of data, appropriate resources, and training for healthcare professionals also poses distinct issues. We propose an approach to identify and treat victims of human trafficking in the ED, with a focus on rural EDs. This approach includes improving data collection and availability on local patterns of trafficking, improving clinician training in identification, and care of victims using trauma-informed techniques. While this case illustrates unique features of human trafficking in the Appalachian region, many of these themes are common to rural areas across the US. Our recommendations emphasize strategies to adapt evidence-based protocols, largely designed in and for urban EDs, to rural settings where clinicians may be less familiar with human trafficking.

## INTRODUCTION

Victims of human trafficking are likely to interact with the healthcare system at some point while they are being trafficked, particularly in the emergency department (ED). One study noted that up to 60–88% of trafficked persons surveyed had visited an ED while actively being trafficked.^1^,^2^ The frequency of ED visits by victims places emergency physicians in a unique position to intervene. Unfortunately, many clinicians lack familiarity and confidence with identifying and caring for victims of trafficking.^3^ In fact, one study showed that less than 5% of emergency physicians felt confident identifying trafficked persons who present to the ED.^4^

This uncertainty may be especially prevalent in rural areas where clinicians perceive trafficking as an urban problem and may lack dedicated training in screening and emergency care of victims of human trafficking. In this article we sought to examine human trafficking in Appalachia to highlight common themes in the emergency care of victims of trafficking, spotlight important issues in rural trafficking including familial trafficking, and explore the vulnerability of the region and marginalized groups living in Appalachia, a region made up of 423 counties across 13 states that spans 205,000 square miles from southern New York to northern Mississippi (Figure). We begin by presenting a real ED case as a stimulus for learning about human trafficking. Then we present realities of Appalachian human trafficking as opposed to common misperceptions. Finally, we conclude with an approach to identifying and treating victims of human trafficking in the ED.

### Case

A 30-year-old female presented to the ED accompanied by police for altered mental status. Per police report, the patient was running in the woods. Her behavior was erratic, and she was unable to provide a succinct history. She endorsed visual hallucinations and lacked focus during the interview. Her vital signs were within normal limits. She appeared anxious but did not have any focal physical exam abnormalities. The patient revealed that she was forced to perform commercial sex acts by her dealer after a recent relapse, had been forced to take illicit drugs, and alluded to physical and sexual assault.

“You’ll meet somebody who will act like some kind of Prince Charming, and they wind up selling you.”

― “Marie,” former sex worker, Charleston, WV.^5^

Human trafficking is defined as the “the act of compelling or coercing a person’s labor, services, or commercial sex acts.”^6^ There are more victims today than at any other time in history, with an estimated 40.3 million victims of human trafficking globally and 24.9 million people trapped in forced labor.^2^ While 16,658 victims were identified in the United States in 2020, it is estimated that 199,000 incidents of trafficking occur in the US every year.^7^,^8^ The US Department of Justice (DOJ) estimates that trafficking generates nearly $150 billion in profits annually.^9^ It is the second largest and fastest growing organized crime trade in the world, recently surpassing the illegal arms trade, and is anticipated to surpass the illegal sale of drugs in the next few years.^10^ Human trafficking occurs in all 50 states and the District of Columbia. There is no typical victim, although marginalized individuals such as homeless youth and those in extreme poverty are at especially high risk.^11^,^12^ Traffickers exploit vulnerabilities such as poverty, addiction, or lack of agency to compel victims into forced labor, commercial sex work, or other activities against their will.^13^,^14^

Appalachia is home to over 26 million residents.^15^ Appalachia’s history is characterized by economic depression, generational poverty, geographic isolation and, more recently, by the devastating impact of the opioid epidemic, all of which are risk factors for human trafficking^5^,^16^ (Table 1). Contrary to common perception among Appalachian residents, human trafficking is not only an urban issue.^16^,^17^ Appalachian states comprise three of the top 10 states for human trafficking with Mississippi, Georgia, and Ohio ranking second, fourth, and fifth, respectively.^8^ Since Appalachia became a battleground for the “war on poverty” in 1965, the region has seen economic gains, although it still lags behind other areas of the country. Between 2015–2019, the median income in Appalachia was 85% the national median. In the same period, the overall poverty rate in Appalachia was 15.2% compared to 13.4% for the US overall; however, the poverty rate in the central subregion was 23.5%.^16^,^18^ Amidst the opioid crisis, which has been responsible for the greatest loss of life of any overdose epidemic, the Appalachian region stands above all others. In this highly rural 13-state region, overdose deaths among those aged 25–44 are over 70% higher than the rest of the US.^19^,^20^

#### Trafficking in Appalachia

A common misperception in the region is that human trafficking occurs only in urban areas and is perpetuated by strangers. In contrast, much of the trafficking in Appalachia is familial, meaning that victims are trafficked by family members, often in exchange for drugs or money.^22^,^23^ In 2013, a survey was conducted to assess professionals who work with minors who were victims of sex trafficking in Kentucky. Most professionals surveyed found that at least one of the victims they had worked with were recruited or lived in Kentucky while being trafficked.^21^ Furthermore, victims who are recruited in Appalachian states may be transported and trafficked in larger, urban areas outside the region. In the same study, two in five professionals stated that at least one of the victims with whom they had worked had been trafficked in states other than Kentucky.^24^ In the case presented above, the victim was both recruited and trafficked within Appalachia prior to her ED presentation.

Based on these studies, it is important for emergency clinicians to stay vigilant and maintain a high suspicion for human trafficking regardless of the patient’s place of origin or current location. Law enforcement personnel in Appalachia note that much of the trafficking is familial and that the practice is very likely severely under-reported.^24^ In one study, up to 44% of data samples included survivors who had been sex trafficked by family members, mainly parents, and most often mothers. Younger girls in rural areas are more likely to be sex trafficked by their parents than those in urban areas, and at younger ages.^25^ From the limited existing data, familial trafficking is more common in rural areas. In a study of 40 adjudicated juvenile females in a southern, rural state, of those trafficked all the rural victims were trafficked by family members; in urban areas, none were trafficked by family members.^25^ In the study from Kentucky, the most mentioned trafficker-victim relationship was family (61.9%).^24^ [It is unknown whether the victim in this case was ever trafficked by family members.] Therefore, a patient who presents with their family should not be assumed to be safe from trafficking and should be screened privately and offered intervention if there is concern for trafficking.^26^

The geography of Appalachia makes it particularly vulnerable to trafficking as well as movement of victims across long distances in a short time. In addition to its rurality, major interstate highways connecting large cities crisscross the region. These highways, thoroughfares of cross-national shipping, bring drugs and buyers into the region and ship men and women out into the commercial sex and labor market. Victims are trafficked along the nation’s highways at truck stops, gas stations, and rest areas.^27^,^28^ In the case we discuss, the victim presented to an ED over 90 miles from her most recent known address within days of being trafficked.

Marginalization and discrimination compound existing vulnerabilities, and those who identify with multiple vulnerable groups are subject to higher risk. Sexual and gender minorities are especially vulnerable in Appalachia where there is an overall lack of LGBTQIA+ specific resources. This is especially true for transgender individuals who are particularly vulnerable to exploitation.^29^ Persons from these groups often struggle to maintain stable employment due to discrimination in the workplace and rely almost exclusively on family and community networks for support.^30^ Unfortunately, as noted above, familial trafficking may compound this exploitation risk. Housing insecurity is also often exploited by traffickers, making homeless persons even more vulnerable. Transgender individuals are more likely to be homeless than their cisgender counterparts. Transgender individuals in the commercial sex industry also face higher rates of violence, with trans women of color facing the highest rates of any group.^29^,^30^ Children who have experienced trauma are also more likely to be trafficked, making those in the foster care system particularly vulnerable. Furthermore, children in foster care may have unmet basic needs.^31^ The opioid epidemic has increased the number of children in foster care nationwide and especially in Appalachia, a problem further exacerbated by the COVID-19 pandemic.^32^,^33^

The overall paucity of data is one of the primary challenges in combating human trafficking worldwide. The data from Appalachia is even less robust than from other areas of the country. In Appalachia, the lack of data means that perhaps even fewer victims than is typical are being identified.^14^ Overall, the DOJ reports that fewer than 1% of victims of human trafficking are identified because of the frequent movement of victims, victims’ inability to escape, and knowledge deficit among healthcare professionals related to the red flags of trafficking.^6^ Up to 88% of victims report accessing healthcare at some point during their trafficking situation, with many presenting to the ED.^2^ Given this, emergency physicians should be extensively trained in identification and intervention for victims of human trafficking (Table 2). Below, we propose recommendations on these practices with particular emphasis on rural settings where clinicians may be less familiar with human trafficking.

##### Case (continued)

In our case, the clinicians were familiar with the signs and symptoms of human trafficking and recognized how these played a significant role in our patient’s clinical presentation. They were able to gain the patient’s trust and reconnect her with resources and a safe house. Unfortunately, despite all efforts from healthcare professionals and ancillary staff, our patient still did not get fully connected to the resources she needed. On chart review, it appears that she presented to another local hospital three days after discharge for medical clearance for jail for methamphetamine use and “engaging in prostitution.” The patient was seen again one month later for medical clearance for a human trafficking program; however, it does not appear that she was directly reconnected with the program and was ultimately discharged and told to follow up as an outpatient. This was her last known encounter with the healthcare system to date.

## RECOMMENDATIONS

A recently published article aptly noted that emergency physicians must educate themselves on the unique aspects of human trafficking in their local area and the resources available to victims.^34^ This is especially pertinent in the Appalachian region given the prevalence of familial trafficking, which is more common than in other regions of the US. Tools such as the HEAL Trafficking Toolkit and Rapid Appraisal for Trafficking (RAFT) screening tool are excellent starting points for developing an ED screening and response protocol; however, protocols must be adjusted to accommodate regional differences^35^,^26^ (Table 3). Ideally, protocols would be evidence-based, but as we have seen with the COVID-19 pandemic, it is not always possible to wait to develop a response until more information becomes available, especially given the overall dearth of data in Appalachia. Furthermore, much of the existing evidence and recommendations come from large urban EDs and are not tailored for rural emergency clinicians. Therefore, improving national and regional data collection on human trafficking must occur in tandem with developing locally tailored systems and protocols for screening and response.

Clinicians looking to create a screening and response protocol in their ED would benefit from collaboration with social work to identify community partners working to combat human trafficking. These partners can help to build a local database of relevant resources for patient referrals and linkage to care. Use of the National Human Trafficking Hotline’s “Framework for a Human Trafficking Protocol in Healthcare Settings” is a useful resource when creating a protocol.^36^ Their website can also be a helpful resource in identifying relevant federal and local laws, as well as potential community partners. Additionally, their website provides educational materials regarding recognition of human trafficking, which can be used for clinician training. While the toolkits mentioned above provide evidence-based screening questions, clinicians implementing these toolkits will still want to ensure that their colleagues have been provided with the education to recognize common signs of human trafficking, such as patients being accompanied by someone who does not let them speak for themselves, patients not being in control of their own legal and financial documents, or tattoos that the patient does not wish to discuss, among others.

Rural clinicians may encounter pediatric and adult victims of human trafficking but have fewer resources to support care of these patients. Mandatory reporting laws for human trafficking vary by state; therefore, clinicians should familiarize themselves with their local policy.^37^ Social workers can assist in providing appropriate care and resources to these patients. In rural areas where resources, including social work, may not be available, clinicians should have a low threshold to discuss with and potentially transfer patients to the nearest referral center where forensic nursing and/or social work support are available. This is especially true for pediatric patients, as dedicated pediatric hospitals may offer significantly more resources for follow-up and ongoing support than rural or critical access hospitals.

Healthcare professionals should be educated on trauma-informed care, as well as on trafficking patterns in their region.^38^ While training ideally begins in undergraduate medical education, it is important that it be consistently accessible to clinicians in various practice environments and throughout the spectrum of practice. The National Human Trafficking Training and Technical Assistance Center (NHTTAC) offers resources for continuing education in trauma-informed care for healthcare professionals.^39^ Furthermore, there is significant variability in the background and training among clinicians, particularly among those working in rural EDs, and those who trained in the era before human trafficking was regularly incorporated in medical education curricula. Therefore, there are likely differences in the degree of formal training regarding identification and care of victims of human trafficking. Additionally, clinicians should have an awareness that due to the fluid nature of human trafficking, they may encounter a patient who does not fit the typical or expected pattern of trafficking within their local community. All clinicians should be educated on resources that are available at their facilities and in their communities, as well as how to connect patients to these services.

## CONCLUSION

Human trafficking victims in Appalachia remain a particularly vulnerable population for which there is little accurate data. Educational strategies can dispel myths and help accurately identify victims. Leveraging and strengthening networks of existing community resources is paramount to combating human trafficking. Finally, improving the availability of data about trafficking from the Appalachian region is crucial to understanding the extent of the problem. Understanding is the first step to identifying, supporting, and protecting the victims and potential victims of trafficking in the Appalachian region.

## Figures and Tables

**Figure:**
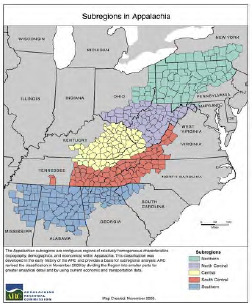
“Appalachia” most commonly refers to the 423-county region designated by the Appalachian Regional Commission (ARC) in 1965, which is divided into 5 subregions. It is important to note that the counties designated by the ARC were included for a variety of reasons, some geographic, some economic, and some political. However, given that lines were not drawn by social ethnographers, there may be persons in areas near the region who identify as Appalachian and whose lived experiences mirror those within the region.^14^,^21^ Map produced by the Appalachian Regional Commission. Used with permission from the ARC.

**Table 1:** Intersectional challenges in Appalachia.

Lack of awareness among the community
Lack of training among healthcare and law enforcement personnel
Lack of resources, including lack of transportation and inadequate funding
Familial trafficking
Poverty
Substance abuse and the opioid epidemic
Cultural differences including traditional gender roles
Truck stop proximity and large network of highways

**Table 2:** Recommendations for emergency clinicians.

Emergency physicians should become familiar with patterns of human trafficking in their area.^34^ Clinicians should recognize that victims may present after being trafficked from a different geographical area and may face unique challenges related to the area from which they were trafficked. Healthcare professionals should advocate for the collection of quantitative data on human trafficking to advance research efforts. This may include multidisciplinary approaches with involvement of health departments, law enforcement, government officials, and other community advocates to add to the fund of available knowledge. Despite the paucity of available data, emergency physicians should strive to create standardized protocols to identify and treat victims of human trafficking in the emergency department. These protocols should be tailored to account for regional differences in trafficking patterns. Clinicians should employ the principles of trauma-informed care when caring for victims of trafficking.^37^ Considerations for care include 1) not having the victim repeat the story so many times; 2) establishing a code word if they feel uncomfortable and want to stop at any point of the exam; and 3) not undressing the patient unless absolutely necessary.^37^ See NHATTC website for further recommendations and suggestions.^39^ Special considerations for rural emergency clinicians are to become familiar with mandatory reporting laws in their area^40^ and consider transfer if a safe discharge plan cannot be established (i.e., resources unavailable).

*NHATTC*, National Human Trafficking and Technical Assistance Center.

**Table 3:** Resources for clinicians in the emergency department.

HEAL protocol toolkit^35^
National Human Trafficking Hotline Awareness materials^38^
HEAL: Legal requirements for reporting^40^
RAFT screening tool^26^
National Human Trafficking Resource Center: Framework for a human trafficking protocol in healthcare settings^36^
